# Stridulating Species of Aphids of the Genus *Uroleucon* (Hemiptera: Aphididae) with Descriptions of a New Species from Iran †

**DOI:** 10.3390/insects16010068

**Published:** 2025-01-12

**Authors:** Mariusz Kanturski, Shalva Barjadze, Andżela Glumac, Natalia Kaszyca-Taszakowska

**Affiliations:** 1 Institute of Biology, Biotechnology and Environmental Protection, Faculty of Natural Sciences, University of Silesia in Katowice, ul. Bankowa 9, 40-007 Katowice, Poland; nitepak@gmail.com (A.G.); natalia.kaszyca-taszakowska@us.edu.pl (N.K.-T.); 2 Institute of Zoology, Ilia State University, Giorgi Tsereteli 3, Tbilisi 0162, Georgia; shalva.barjadze@yahoo.com

**Keywords:** new taxon, aphid, *Asyneuma*, *Michauxia*, Middle East

## Abstract

In this paper, we present a review of stridulating species of aphids of the Macrosiphini genus *Uroleucon* (Hemiptera, Aphididae) and include 13 species. The most characteristic feature of the reviewed species is the presence of unusual aphids’ peg-like sensilla distributed on the hind tibia, which are rubbed against the surface of the siphunculi. A new aphid species—*Uroleucon remaudierei* **sp. nov.**, living on *Asyneuma persicum* and *Michauxia laevigata* (Campanulaceae) in Iran—is described and illustrated based on the apterous and alate viviparous females. The new species is differentiated from other morphologically similar congeners. An identification key for apterous viviparous females of all *Uroleucon* species living on Campanulaceae and sound-producing species of the genus *Uroleucon* are provided. Additionally, a scanning electron microscopy analysis of the apterous and alate viviparous females of this species was made to elucidate the features of the general morphology, antennal sensilla, and stridulatory apparatus for the first time.

## Simple Summary

This publication shows a comparative review of aphid species of the genus *Uroleucon* characterized by the presence of short, peg-like setae on the hind tibia, which most probably are used during sound production with a description of a new aphid species. We used scanning electron microscopy (SEM) to show the morphology (gross morphology, sensilla, and the stridulatory apparatus) of the representative of the genus *Uroleucon* for the first time.

## 1. Introduction

The genus *Uroleucon* Mordvilko, 1914, which comprises 258 aphid species in the world united into six subgenera, is one of the most speciose genera in the tribe Macrosiphini Wilson, 1910 [1]. The genus is generally characterized by a smooth head, well-developed antennal tubercles, the presence of secondary rhinaria on the antennal segment III in apterae, very long processus terminalis, ultimate rostral segments with blunt apices, long and cylindrical reticulated siphunculi, a lanceolate cauda with a pointed apex, and the first tarsal segments with five or rarely three or four setae. In many species, the abdominal dorsal setae are located on pigmented scleroites [2,3]. Most species live on herbaceous plants that belong to the families Asteraceae (at least 200 species) and Campanulaceae (11 species) without any host alternation, while the remaining species utilize host plants from other plant families [2,3].

Thirty two species of *Uroleucon*, *U*. *achilleae* (Koch), *U*. *acroptilidis* Kadyrbekov, Renxin and Shao, *U*. *aeneum* (Hille Ris Lambers), *U*. *bielawskii* (Szelegiewicz), *U*. *campanulae* (Kaltenbach), *U*. *carthami* (Hille Ris Lambers), *U*. *caspicum* Rezwani and Lampel, *U*. *chondrillae* (Nevsky), *U*. *cichorii* (Koch), *U*. *cirsii* (L.), *U*. *compositae* (Theobald), *U*. *elbursicum* Lampel and Rezwani, *U*. *erigeronense* (Thomas), *U*. *favreti* Mehrparvar & Kadyrbekov, *U*. *helichrysi* Nieto Nafría and Pérez Hidalgo, *U*. *hymenocephali* Rezwani and Lampel, *U*. *hypochoeridis* (Hille Ris Lambers), *U*. *inulae* (Ferrari), *U*. *iranicum* Holman, *U*. *jaceae* (Linnaeus), *U*. *loxdalei* Mehrparvar, Rakhshania, Rokni and Kanturski, *U*. *murale* (Buckton), *U*. *ochropus* (Hille Ris Lambers), *U*. *picridis* (Fabricius), *U*. *pilosellae* (Börner), *U*. *rapunculoidis* (Börner), *U*. *sonchi* (Linnaeus), *U*. *tanaceti* (Linnaeus), *U*. *taraxaci* (Kaltenbach), *U*. *teheranense* Nieto Nafría and Pérez Hidalgo, *U*. *tortuosissimae* Rezwani and Lampel, and *U*. *tussilaginis* (Walker) have hitherto been recorded in Iran [3,4,5,6,7].

The phenomenon of stridulation in aphids has been described for the first time in *Aphis* (*Toxoptera*) *aurantii* Boyer de Fonscolombe, 1841 [8], and later reported or described by Eastop [9,10] but referred to by Aphidini. Later, Holman [11] stated that similar structures and possible stridulation may occur in other genera in the tribe Macrosipini. Holman (1994) pointed out that structures, like rows of short, peg-like setae, which may be responsible for the production of sound, can be found in the genera *Macrosiphoniella*, *Sitobion*, and *Uroleucon*, in which the vast majority of sound-producing species may be found.

During an examination of Georges Remaudière’s aphid collection in the Muséum National d’Histoire Naturelle, Paris (MNHN), and Jaroslav Holman’s collection in the Biology Centre, Czech Academy of Sciences, České Budějovice, slides with *Uroleucon* sampled on *Asyneuma persicum* Bornm. and undetermined *Michauxia* L’Hér. (as *Mindium* sp. on slides) (Campanulaceae) in Iran were found, and a new species was recognized. The new taxon has a row of short, thick peg-like setae on the hind tibia ventrally, like other known species in the genus *Uroleucon*, which also can produce stridulation in the environment [11,12]. In this paper, we present a review of the stridulating species of the genus *Uroleucon* and compare it with the new species. Additionally, we provided a scanning electron microscopy analysis of the morphological characters, sensilla, and the stridulating apparatus for the first time.

This paper is a part of more detailed research on the morphology of the stridulating apparatus in aphids as well as the taxonomy and phylogeny of the aphid genus *Uroleucon*.

## 2. Materials and Methods

### 2.1. Material and Taxonomy

The specimens were examined using a Leica DM 3000 LED light microscope and photographed using a Leica MC 190 HD camera using a differential interference contrast. The measurements were taken according to Ilharco and van Harten [13]. All of the measurements are given in millimeters. The current host plant names are given according to World Flora Online [14].

The following abbreviations are used: **ABD TERG**: abdominal tergite; **ANT**: antennae or their lengths; **ANT I**–**VI**: antennal segments I, II, III, IV, V, and VI or their lengths (ratios between antennal segments are simply given as, e.g.**,** ‘VI: III’); **BASE**: basal part of last antennal segment or its length; **BD III**: basal articular diameter of ANT III; **BL**: body length (from the anterior border of the head to the end of cauda); **HT I**: first segment of the hind tarsus; **HT II**: second segment of the hind tarsus or its length; **HW**: greatest head width across the compound eyes; **LS ANT III**: length of the longest setae of ANT III; **PT**: processus terminalis of the last antennal segment or its length; SIPH: siphunculus or its length; **III FEMORA**: hind femora length; **III TIBIAE**: hind tibiae length; and **URS**: ultimate segments of the rostrum (IV + V) or their lengths. In the case of a series of single slides with a single specimen with the same collection data for the examined material sections, all of them present the same data as the full previous slide in order to avoid repetition.

The type material is deposited in the **DZUS**—Zoology Research Team, University of Silesia in Katowice Hemiptera Collection, Katowice (Poland), the **IECA**—the Biology Centre of the Czech Academy of Sciences, Institute of Entomology, České Budějovice (Czech Republic), the **IZISU**—the Institute of Zoology, Ilia State University, Tbilisi (Georgia), and the **MNHN**—the Muséum national d’Histoire naturelle, Paris (France).

### 2.2. Scanning Electron Microscopy

Apterous viviparous females for this study came from the alcohol-preserved samples deposited in the IECA. Dehydration was accomplished through an ethanol series of 80%, 90%, 96%, and two changes of dilute ethanol for 10 min each. Absolute ethanol-dehydrated specimens were treated with chloroform for 48 h. Dehydrated and cleaned samples were dried using the Leica EM CPD 300 auto-critical point dryer (Leica Microsystems, Vienna, Austria). Dry samples were mounted on aluminum stubs with double-sided adhesive carbon tape and sputter coated with a 45 nm gold layer in a Quorum 150 T ES Plus sputter coater (Quorum Technologies Ltd., Laughton, East Sussex, UK). The specimens were imaged by the Hitachi SU8010 field emission scanning electron microscope FESEM (Hitachi High-Technologies Corporation, Tokyo, Japan) at 5, 7, and 10 kV accelerating voltage with a secondary electron detector (ESD). Final figure processing was performed using PhotoScape 3.7 (photoscape.org, accessed on 28 October 2024) and IrfanView 64 (irfanview.com, accessed on 20 October 2024).

## 3. Results

### 3.1. Taxonomy

#### Review of Stridulating Species of the Aphid Genus *Uroleucon*

1. ***Uroleucon (Uromelan) adenophorae*** (Matsumura, 1918)

*Macrosiphum adenophorae* Matsumura, 1918 [15]: 3

**Figure 1:**
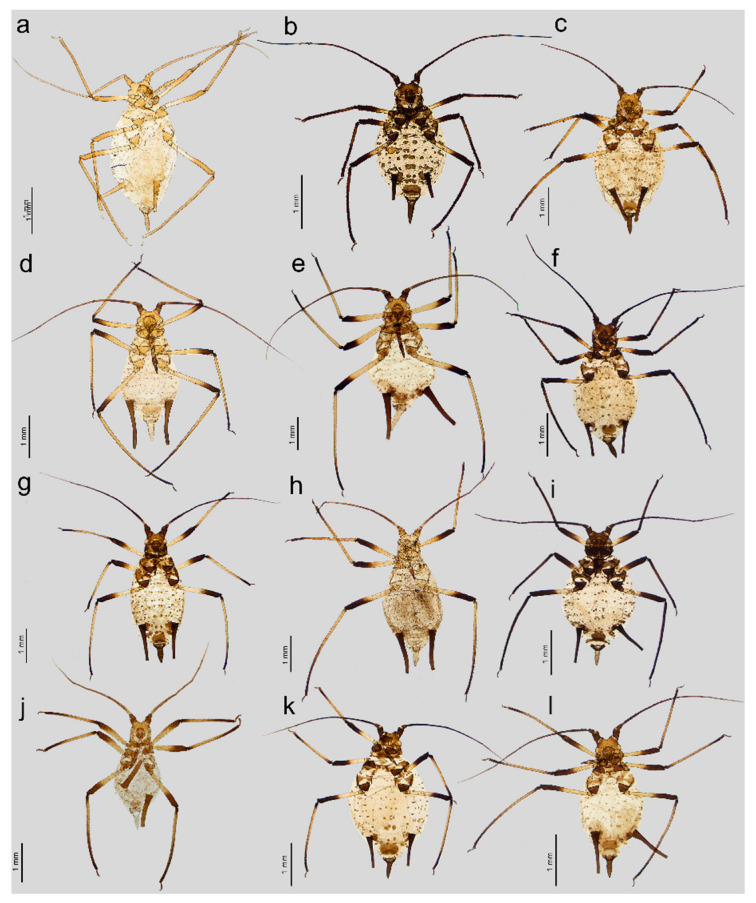
General view of apterous viviparous females of stridulating *Uroleucon* species: (**a**) *U*. *adenophorae*, (**b**) *U*. *campanulae*, (**c**) *U*. *carthami*, (**d**) *U*. *caspicum*, (**e**) *U*. *cirsicola*, (**f**) *U*. *jaceae*, (**g**) *U*. *minor*, (**h**) *U*. *monticola*, (**i**) *U*. *phyteuma*, (**j**) *U. remaudierei* **sp. nov.**, (**k**) *U*. *riparium*, (**l**) *U*. *siculum*.

**Figure 2:**
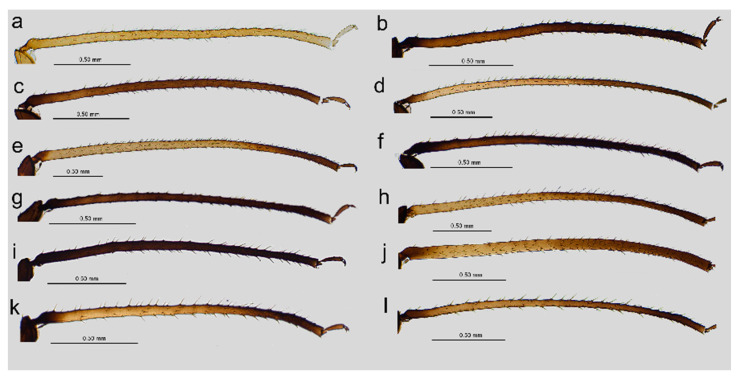
III TIBIAE pigmentation of apterous viviparous females of stridulating *Uroleucon* species: (**a**) *U*. *adenophorae*, (**b**) *U*. *campanulae*, (**c**) *U. carthami*, (**d**) *U*. *caspicum*, (**e**) *U*. *cirsicola*, (**f**) *U*. *jaceae*, (**g**) *U*. *minor*, (**h**) *U*. *monticola*, (**i**) *U*. *phyteuma*, (**j**) *U*. *remaudierei* **sp**. **nov**., (**k**) *U*. *riparium*, (**l**) *U*. *siculum*.

**Figure 3:**
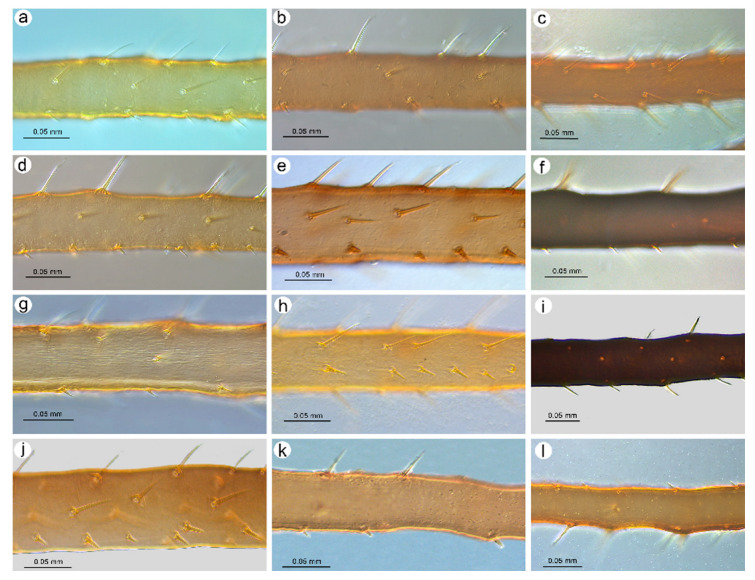
Sense pegs on the inner side of III TIBIAE of apterous viviparous females of stridulating *Uroleucon* species: (**a**) *U*. *adenophorae*, (**b**) *U*. *campanulae*, (**c**) *U*. *carthami*, (**d**) *U*. *caspicum*, (**e**) *U*. *cirsicola*, (**f**) *U*. *jaceae*, (**g**) *U*. *minor*, (**h**) *U*. *monticola*, (**i**) *U*. *phyteuma*, (**j**) *U*. *remaudierei* **sp**. **nov**., (**k**) *U*. *riparium*, (**l**) *U*. *siculum*.

**Figure 4:**
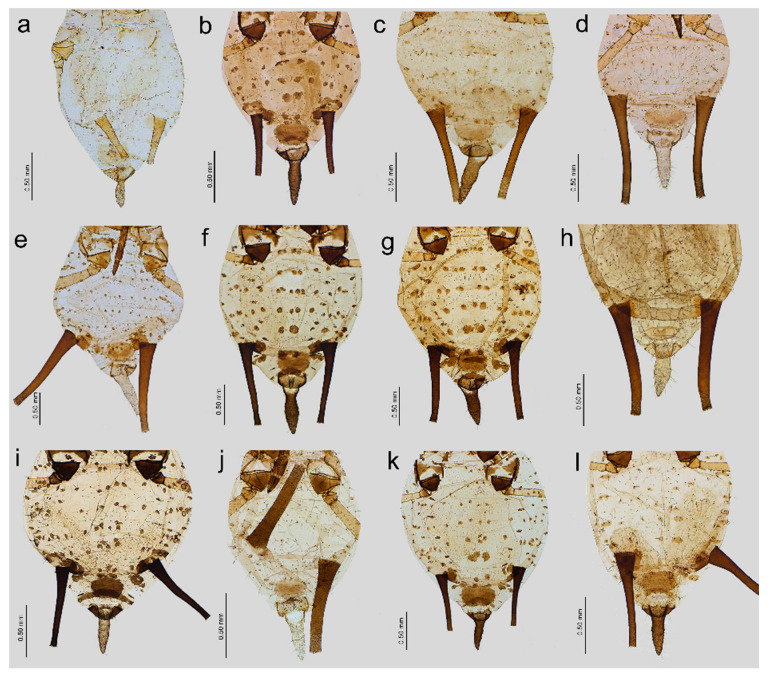
Characters of abdomen of apterous viviparous females of stridulating *Uroleucon* species: (**a**) *U*. *adenophorae*, (**b**) *U*. *campanulae*, (**c**) *U*. *carthami*, (**d**) *U*. *caspicum*, (**e**) *U*. *cirsicola*, (**f**) *U*. *jaceae*, (**g**) *U*. *minor*, (**h**) *U*. *monticola*, (**i**) *U*. *phyteuma*, (**j**) U. *remaudierei* **sp**. **nov**., (**k**) *U*. *riparium*, (**l**) *U*. *siculum*.

**Apterous viviparous female**. Color in life: shiny dark brown to black [2]. Morphological characters: Head slightly sclerotized, light brown, ANT uniformly light brown, femora light brown with pale distal halves, and tibiae light brown with slightly paler inner distal parts (Figure 1a). Peg-like setae on the inner side of the tibiae are rather long, similar to the other setae, and pointed (Figure 2a and Figure 3a). Abdomen pale with light brown to brown scleroites, light brown SIPH, postsiphuncular sclerites, and cauda (Figure 4a).
**Host plant**: Species of *Adenophora* and *Campanula expansa* (=*Astrocodon kruhseanus*) (Campanulaceae) [3,16].
**Distribution**: Japan (*terra typica*), Mongolia, the Republic of Korea, and the Russian Federation: Far Eastern Federal Districts [3,16,17].

2. ***Uroleucon (Uromelan)* campanulae** (Kaltenbach, 1843)

*Aphis campanulae* Kaltenbach, 1843 [18]: 26

**Apterous viviparous female**. Color in life: They are rather shiny with a dark brown head and pronotum and are brown on the rest of their body with dark ANT. Femora are dark brown to black with yellow to pale proximal halves. Tibiae, SIPH, and cauda dark brown to black.

Pigmentation on slide: Head sclerotized, brown. ANT dark brown with slightly lighter ANT VI. Thorax sclerotized, brown. Femora dark to black with yellow proximal halves, and tarsi dark (Figure 1b). Tibiae are generally dark brown, sometimes with a lighter proximal part (Figure 2b). Peg-like setae are short and slender with slightly rounded apices (Figure 3b). Abdomen yellow with well-developed sclerotization in the form of quite big brown scleroites at setal bases of which the slinal ones are bigger than the rest. SIPH with ante- and postsiphucular sclerites, cauda brown (Figure 4b).

**Host plant**: Species of *Campanula* and *Jasione* (Campanulaceae) [3,17].
**Distribution**: This is a common Palaearctic species that is rather widely distributed in Europe (with Germany as the *terra typica*), western Siberia, and southwest and central Asia [3].

3. ***Uroleucon*** (***Uromelan***) ***carthami*** (Hille Ris Lambers, 1948)

*Dactynotus* (*Uromelan*) *carthami* Hille Ris Lambers, 1948 [19]: 276

**Apterous viviparous female**. Color in life: shiny blackish-brown or almost black with only femora pale on the proximal halves. Pigmentation on slide: Head sclerotized, brown. ANT brown with slightly lighter very basal part of ANT III. Pronotum sclerotized, brown. Femora dark with yellow proximal halves. Fore and middle tobiae yellow to light brown with darker proximal and distal ends. (Figure 1c). III TIBIAE brown with a lighter proximal part (Figure 2c). Peg-like setae of different lengths, from short to long, with slightly blunt and pointed apices (Figure 3c). Abdomen yellow with well-developed scleroites at setal bases, which all are of the same size. SIPH with postsiphucular sclerites, cauda brown (Figure 4c).
**Host plant**: Species of *Carthamus* (Asteraceae) [3,17].
**Distribution**: *Uroleucon carthami* is distributed in Great Britain and southern and central Europe. In addition to Europe, this species was observed in Algeria, Israel (*terra typica*), Lebanon, Turkey, Iran, Kazakhstan, Pakistan, and India (Kashmir) [3,17,20,21].

4. ***Uroleucon (Uroleucon) caspicum*** (Rezwani and Lampel, 1990)

*Uroleucon* (*Uroleucon*) *caspicum* Rezwani and Lampel, 1990 [22]: 245

**Apterous viviparous female**. Color in life: shiny dark brown with dark ANT, legs, and SIPH. Cauda pale [22]. Pigmentation on slide: Head sclerotized, light brown, and thorax rather membranous. ANT brown with lighter basal part of ANT III. Femora yellow with brown distal ends. Fore and middle tibiae light brown with brown distal and proximal ends (Figure 1d). III TIBIAE yellow with dark knee area and proximal halves (Figure 2d). Peg-like setae short, fine, and pointed (Figure 3d). Abdomen pale with poorly visible scleroites at setal bases. SIPH uniformly brown with very poorly visible postsiphuncular sclerites. Cauda pale (Figure 4d).
**Host plant**: *Klasea* (=*Serratula*) *quinquefolia* (Asteraceae) [3].
**Distribution**: Distributed in Iran (*terra typica*) [22] and the Russian Federation (North Caucasus Federal District) [23].

5. *Uroleucon (Uroleucon) cirsicola* (Holman, 1962)

*Dactynotus cirsicola* Holman, 1962 [24]: 32

**Apterous viviparous female**. Color in life: dark brassy brown, with often black ANT, legs mainly pale brown, and SIPH black on basal half but brown distally. Cauda yellow (Holman, 1962). Pigmentation on slide: Head sclerotized, prothorax, and mesothorax sclerotized and brown. ANT brown with darker ANT I and II and lighter basal part of ANT III. Femora yellow with dark distal ends. Fore and middle tibiae yellow with brown distal and proximal ends (Figure 1e). III TIBIAE yellow with brown knee area and proximal end (Figure 2e). Peg-like setae very short, slightly bulky at bases with rounded apices (Figure 3e). Abdomen pale with well-visible small scleroites at setal bases. SIPH brown with slightly lighter apical ends and well-developed postsiphuncular sclerites. Cauda pale (Figure 4e).
**Host plant**: Species of *Arctium*, *Cirsium*, *Senecio*, and *Tragopogon* (Asteraceae) [17].
**Distribution**: Crimea in Ukraine (*terra typica*) [24] and recorded also in Moldova, Turkey, Georgia, and the Russian Federation: western Siberia [3,17,25].

6. Uroleucon (Uroleucon) fuchuense (Shinji, 1942)

*Macrosiphum fuchuensis* Shinji, 1942 [26]: 4

**Apterous viviparous female**. Color in life: Body shiny red with dark ANT, tibiae, and SIPH. Femora black with pale proximal parts. Cauda pale with slightly dusky very distal end [27]. Pigmentation on slide: Head sclerotized, brown. ANT dark, almost black. Femora yellow with dark to black distal halves. Tibiae yellow to light brown with dark knee areas and distal halves. Peg-like setae short and very short, but the difference in size is visible, straight with slightly pointed apices. Abdomen pale with small indistinct scleroites at setal bases, dark brown SIPH, and pale brown cauda.
**Host plant**: Species of *Aphillea*, *Artemisia*, *Aster*, *Cacalia*, *Lactuca*, *Picris*, *Pterocypsela*, and *Saussurea* (Asteraceae) [3,17].
**Distribution**: So far, this species has been recorded in Japan (*terra typica*) [26], the Republic of Korea [27], and the Russian Federation: eastern Siberia and Far Eastern Federal Districts [17,28].
**Remarks**. Kanturski and Lee [27] gave detailed descriptions and figured the sense pegs of this species based on oviparous females.

7. ***Uroleucon (Uromelan) jaceae*** (Linnaeus, 1758)

*Aphis jaceae* Linnaeus, 1758 [29]: 452

**Apterous viviparous female**. Color in life: shiny, and the color varies from reddish brown to blackish brown with a visible row of dorsal spots on the abdomen (especially in lighter individuals). ANT are black as well as the legs, in addition to the greenish proximal parts of the femora. SIPH and cauda black. Pigmentation on slide: Head and thorax sclerotized and brown. ANT dark brown to black. Femora yellow with dark distal halves (Figure 1f). Tibiae including III TIBIAE dark to black with only very slightly lighter proximal parts (Figure 2f). Peg-like setae very short, straight with slightly rounded or pointed apices (Figure 3f). Abdomen yellow with well-developed scleroites at setal bases, of which the spinal ones often are larger than the rest. SIPH dark brown with well-developed, dark postsiphuncular sclerites. Cauda brown (Figure 4f).
**Host plant**: Various genera of Asteraceae [3,17].
**Distribution**: *Uroleucon jaceae* is known throughout the western and central Palearctic (with Sweden as the *terra typica*), from the Atlantic islands of Europe (e.g., Madeira) to Siberia and India (Western Himalaya), including territories as far south as Egypt and Saudi Arabia [3,30].

8. ***Uroleucon*** (***Uromelan***) ***minor*** (Börner, 1940)

*Dactynotus minor* Börner, 1940 [31]: 4

**Apterous viviparous female**. Color in life: brown with black dorsal spots, legs yellow banded with black ANT, SIPH, and cauda (Blackman and Eastop, 2024). Pigmentation on slide: Head, pro and mesothorax sclerotized and brown. ANT dark brown to black with lighter basal part of ANT III. Femora yellow with dark distal halves. Tibiae including III TIBIAE yellow or light brown, with the knee area and distal ends dark (Figure 1g) or III TIBIAE dark with very slightly lighter proximal part (Figure 2g). Peg-like setae short, fine, and with clearly pointed apices (Figure 3g). Abdomen yellow with well-developed scleroites at setal bases, of which the spinal ones on ABD V and VI often are larger than the rest. SIPH dark brown with well-developed, dark postsiphuncular sclerites. Some sclerotization can be noted and treated as antesiphuncular sclerites, but those are rather fused spinal and marginal scleroites. Cauda brown (Figure 4g).
**Host plant**: species of *Klasea* and *Tanacetum corymbosum* (Asteraceae) [3,17].
**Distribution**: This Palaearctic species (with Austria as *terra typica*) is rather widely distributed in Europe, east to southwest Siberia in the Russian Federation, and Kazakhstan [3,17,32].

9. ***Uroleucon (Uroleucon) monticola*** (Takahashi, 1935)

*Macrosiphum monticolum* Takahashi, 1935 [33]: 502

**Apterous viviparous female**. Color in life: shiny green, dusky around bases of SIPH, with distal halves of femora and tibiae brown-black, black SIPH, and a contrastingly pale yellow cauda (Blackman and Eastop, 2024). Pigmentation on slide: Head slightly sclerotized, yellow to pale brown. ANT brown with yellow basal parts of ANT I, ANT III, and light brown distal part of ANT V. Femora yellow or light brown with dark distal halves (Figure 1h). Tibiae including III TIBIAE light brown also in the knee area with darker distal halves (Figure 2h). Peg-like setae rather long and robust with slightly bulky basal parts and pointed (Figure 3h). Abdomen pale to yellow with very few indistinct scleroites at setal bases, which in general view are invisible. SIPH brown with lighter postsiphuncular sclerites. Cauda pale or yellow (Figure 4h).
**Host plant**: Species of *Aster*, *Conyza*, and *Erigeron* (Asteraceae) [3,17].
**Distribution**: This East Asiatic species can be found in Taiwan (*terra typica*) [33], Japan [34], the Republic of Korea [35], and China [3].

10. ***Uroleucon (Uromelan) phyteuma*** (Bozhko, 1950)

*Megalosiphum phyteuma* Bozhko, 1950 [36]: 132

**Apterous viviparous female**. Color in life: aphids are slightly shiny and uniformly black (Holman, 1969). Morphological characters: Head sclerotized, brown to dark brown, ANT uniformly brown, femora brown to dark with pale distal parts of fore and middle tibiae and pale proximal halves of III FEMORA (Figure 1i). Tibiae brown to dark uniformly (Figure 2i). Peg-like setae on the inner side of tibiae rather long, similar to the other setae, and pointed (Figure 3i). Abdomen pale with brown scleroites at setal bases and characteristically large marginal tubercles on ABD TERG I-IV. SIPH dark brown with brown postsiphuncular sclerites and fused, which may be confused with antesiphuncular sclerites. Cauda light brown (Figure 4i).
**Host plant**: *Asyneuma canescens* (Campanulaceae) [3,17].
**Distribution**: So far, this species is known in Ukraine (*terra typica*) and the Slovak Republic [36,37].

**Figure 5:**
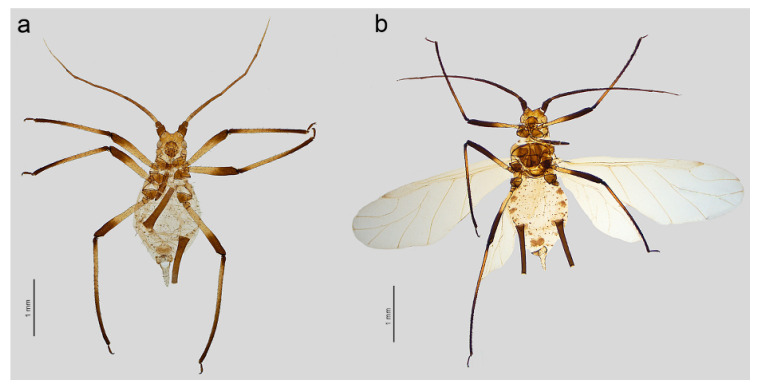
*Uroleucon remaudierei* **sp**. **nov**. general view: (**a**) apterous viviparous female, (**b**) alate viviparous female.

**Figure 6:**
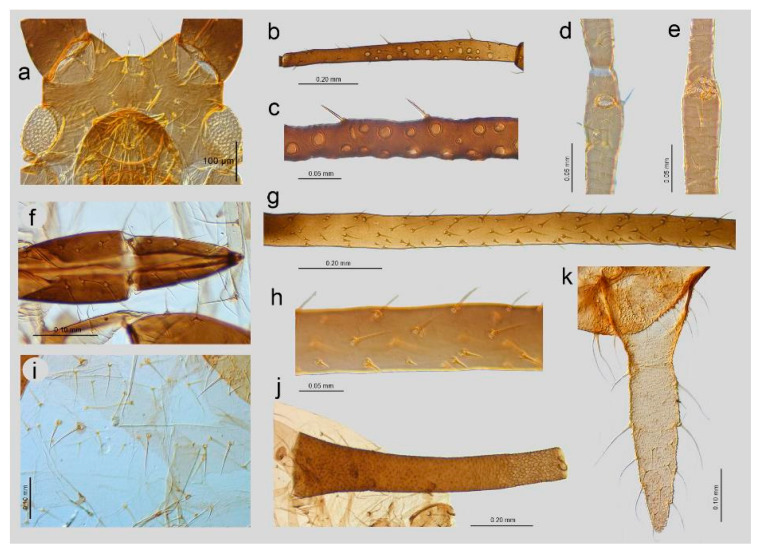
Detailed morphological characters of apterous viviparous female of *Uroleucon remaudierei* **sp**. **nov**.: (**a**) head, (**b**) secondary rhinaria distribution of ANT III, (**c**) structure of rhinaria on ANT III, (**d**) primary rhinarium on ANT V, (**e**) primary rhinaria on ANT VI, (**f**) URS, (**g**) sensilla and sense pegs distribution on III TIBIAE, (**h**) structure of the sense pegs on the inner side of III TIBIAE, (**i**) dorsal abdominal chaetotaxy, (**j**) SIPH, (**k**) cauda.

**Figure 7:**
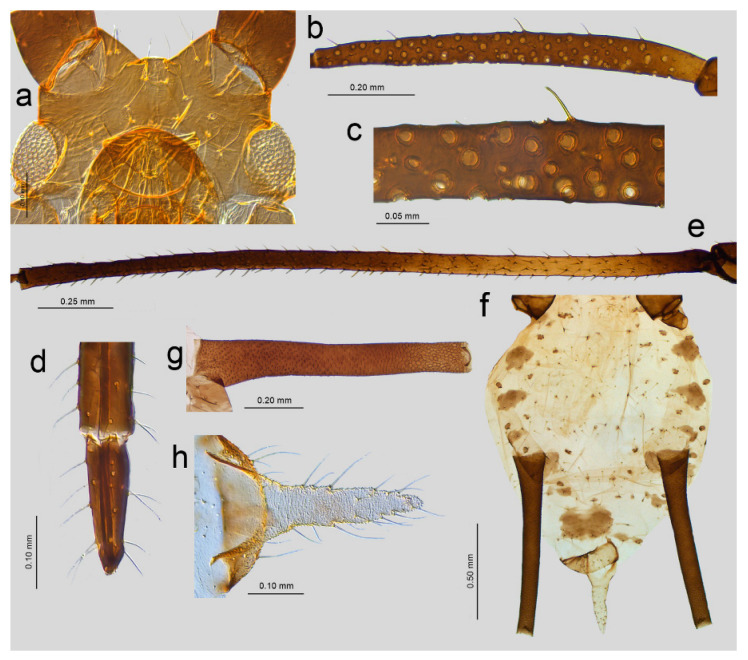
Detailed morphological characters of alate viviparous female of *Uroleucon remaudierei* **sp**. **nov**.: (**a**) head, (**b**) secondary rhinaria distribution of ANT III, (**c**) structure of the secondary rhinaria on ANT III, (**d**) URS, (**e**) sensilla and sense pegs distribution on III TIBIAE, (**f**) abdomen, (**g**) SIPH, (**h**) cauda.

11. *Uroleucon (Uroleucon) remaudierei* sp. nov.

(Figure 1j, Figure 2j, Figure 3j, Figure 4j, Figure 5a,b, Figure 6a–k, and Figure 7a–h, Table 1)

Description. **Apterous viviparous female** (n = 18).

Color in life: unknown; pigmentation on slide: head sclerotized, light brown; ANT brown with lighter basal part of ANT III; rostrum sclerotized, brown; femora light brown with darker distal halves; tibiae brown with darker proximal bases and distal halves, tarsi brown; abdomen yellow with few brown scleroites; and SIPH uniformly brown, cauda yellow (Figure 1j, Figure 2j, and Figure 5a). Morphometric characters: HW 0.17–0.19 × ANT. Head with long, rigid, and pointed setae, 0.050–0.09 mm long. ANT tubercles each with 2–4 setae on internal angles (Figure 6a). ANT 0.87–1.05 × BL. ANT III with 7–37 rounded, different-sized secondary rhinaria with well-developed sclerotized rims (Figure 6b,c), ANT IV always longer than ANT V. ANT V with primary rhinarium surrounded with sclerotic ring with many projections (Figure 6d). ANT VI with tightly adjoining six accessory rhinaria with projections (Figure 6e). PT 3.62–5.18 × BASE. Other antennal ratios: VI:III 0.91–1.32, V:III 0.47–0.60, IV:III 0.59–0.81, PT:III 0.73–1.10, PT:IV 1.10–1.46, and PT:V 1.45–1.84. ANT chaetotaxy: ANT have thick, rigid setae with blunt apices. ANT III setae are 0.03–0.05 mm long, LS ANT III 0.72–1.25 × BD III. ANT III with 21–28, ANT IV with 10–16, and ANT V with 7–11 setae. ANT VI with 3–4 basal, 4 apical, and 4–6 setae along the PT. Rostrum reaching hind coxae. URS 0.19–0.25 × ANT III, 0.17–0.22 × ANT VI, 0.21–0.28 × PT, 0.96–1.13 × BASE, and 0.96–1.06 × HT II with 8–9 fine and pointed accessory setae (Figure 6f). III FEMORA have short to medium-long, stiff, rigid, and rather pointed setae, 0.018–0.050 mm long. Setae on III TIBIAE are rigid, short to medium long, pointed setae, 0.020–0.060 mm long (Figure 6g). Internal side of III TIBIAE with a row of very short, thick peg-like setae, 0.007–0.027 mm long with pointed apices (Figure 3j and Figure 6h). HT I with 5:5:5 setae, HT II 0.20–0.26 × ANT III, 0.18–0.22 × ANT VI, 0.21–0.28 × PT, and 1.00–1.15 × BASE. Abdomen membranous without marginal tubercles with long and rigid setae with pointed apices, 0.050–0.100 mm long on ABD TERG I–V and 0.050–0.125 mm long on ABD TERG VI–VIII (Figure 6i). Only few setae on abdomen arise from small and slightly irregular scleroites. ABD TERG VIII with 5–8 setae. SIPH tubular, tapering, imbricated with a distinct zone of subapical reticulation, without antesiphuncular and postsiphuncular sclerites and without flange (Figure 4j and Figure 6j). Reticulated zone 0.22–0.34 × SIPH. SIPH 1.84–2.18 × cauda, 0.26–0.32 × BL, and 0.92–1.22 × ANT III. Genital plate with 2–4 anterior, 2 median, and 7–11 posterior setae. Cauda lanceolate, 1.90–2.60 × its width at base and 0.14–0.16 × BL with 13–18 long, fine, and pointed setae (Figure 6k). Measurements are available in Table 1.

**Alate viviparous female** (n = 5)

Color in life: unknown; pigmentation on slide: head sclerotized, light brown; ANT dark brown with only slightly paler basal part of ANT III; and rostrum and thorax sclerotized, brown. Femora dark brown with about 1/3 proximal part yellow; tibiae dark brown with lighter proximal part, tarsi dark brown; abdomen yellow with brown marginal sclerites and few scleroites; and SIPH uniformly brown, cauda pale yellow (Figure 5b).

Morphometric characters: HW 0.16–0.17 × ANT. Head with long, rigid, and slightly blunt setae, 0.035–0.070 mm long. ANT tubercles each with 3–4 setae on internal angles (Figure 7a). ANT 0.89–1.07 × BL. ANT III with 57–80 rounded, different-sized secondary rhinaria with well-developed sclerotized rims and distributed on the whole length of the segment (Figure 7b,c), ANT IV always longer than ANT V. ANT V with primary rhinarium surrounded with sclerotic ring with many projections. ANT VI with tightly adjoining accessory rhinaria with projections. PT 4.00–4.10 × BASE. Other antennal ratios: VI:III 0.89–1.02, V:III 0.47–0.59, IV:III 0.62–0.69, PT:III 0.71–0.81, PT:IV 1.03–1.30, and PT:V 1.33–1.71. ANT chaetotaxy: ANT have thick, rigid setae with blunt or very narrow-capitate apices. ANT III setae are 0.035–0.040 mm long, LS ANT III 0.66–0.07 × BD III. ANT III with 24–32, ANT IV with 14–15, and ANT V with 7–10 setae. ANT VI with 4 basal, 4 apical, and 4–6 setae along the PT. Rostrum reaching hind metasternum. URS 0.19–0.21 × ANT III, 0.20–0.21 × ANT VI, 0.25–0.27 × PT, 1.02–1.08× BASE, and 0.89–0.97 × HT II with eight fine and pointed accessory setae (Figure 7d). III FEMORA have short to medium-long, stiff, rigid, and rather pointed setae, 0.020–0.040 mm long. Setae on III TIBIAE are rigid, short to medium long, pointed setae, 0.020–0.050 mm long (Figure 7e). HT I with 5:5:5 setae, HT II 0.21–0.23 × ANT III, 0.21–0.23 × ANT VI, 0.26–0.29 × PT and 1.00–1.18 × BASE. Abdomen membranous without marginal tubercles and with rather long and rigid setae with pointed apices, 0.050–0.075 mm long on ABD TERG I–V and 0.065–0.08 mm long on ABD TERG VI–VIII. Abdomen with marginal sclerites on ABD TERG II–V. Only few setae on abdomen arise from small and slightly irregular scleroites. ABD TERG VIII with 6–8 setae (Figure 7f). SIPH tubular, tapering, and imbricated with a distinct zone of subapical reticulation, with large postsiphuncular sclerites and without flange (Figure 7g). Reticulated zone 0.23–0.28 × SIPH. SIPH 2.36–2.438 × cauda, 0.27–0.34 × BL, and 1.02–1.12 × ANT III. Genital plate with 2 anterior, 2 median, and 8–9 posterior setae. Cauda lanceolate, 1.34–2.15 × its width at base and 0.11–0.13 × BL with 14–15 long, fine and pointed setae (Figure 7h). Measurements are available in Table 1.

**Types**: Holotype. Apterous viviparous female (apt.), Iran, Gajereh, 2300 m, 14.07.1955, *Michauxia leavigata*, G. Remaudière leg., i767 (apt 4), IECA. Paratypes. Apt., other data same as in holotype, i767 (apt 3), IECA; alate viviparous female (al.), other data same as in holotype, i767 (al 3), IECA; apt., other data ast in holotype, 24911, MNHN; apt., other data as in holotype, 24913, MNHN; four apt., Fasham, 1800 m, 08.09.1972, *Mindium laevigatum* (=*Michauxia laevigata*), G. Remaudière leg., i3699 (apt. 23–26), IECA; 2 al., i3699 (al. 7–8), IECA; 2 al., i3699 (al. 9–10), IECA; 2 apt., i3699 (apt. 21–22), IECA; 4 apt., 25 Km E from Sanandaj, 2800 m, 15.08.1955, *Asyneuma persicum*, G. Remaudière leg., i1048 (apt. 9–12), IECA; apt., Kuh-e Dinar, 3300 m, 13.09.1955, i1112 (apt. 14), IECA, IECA; apt., i1112 (apt. 15), IECA; i1112 (apt. 10), DZUS; apt., i1112 (apt. 11–13), ISIZU.
**Diagnosis and taxonomical comments**. The new species belongs to subgenus *Uroleucon* Mordvilko, 1914, with a row of short thick peg-like setae on the hind tibia ventrally. The following species from the same subgenus have a row of short thick peg-like setae: *U*. *caspicum*, *U*. *cirsicola*, *U*. *fuchuense*, and *U*. *monticola*. The apterous viviparous females of *Uroleucon remaudierei* **sp**. **nov**. are distinguished from those of *U*. *caspicum* by (1) the number of setae on the cauda: cauda with 13–18 setae in the new species, while there are 24–41 setae in *U*. *caspicum*; (2) the URS L/HT II L ratio: 0.97–1.07 in the new species and 1.20–1.46 in *U*. *caspicum*; and (3) the SIPH L/BL ratio: 0.27–0.32 in the new species and 0.33–0.44 in *U*. *caspicum* [3,22].

The new species lives on *Asyneuma persicum* and *Michauxia laevigata* in Iran, while *U*. *caspicum* lives on *Serratula quinquefolia* in Iran and Russia (Caucasus) [3].

The apterous viviparous females of *Uroleucon remaudierei* **sp**. **nov**. are distinguished from those of *U*. *cirsicola* by (1) the number of setae on cauda: 13–18 setae in the new species, while there are 30–45 setae in *U*. *cirsicola*; (2) the presence/absence of antesiphuncular sclerites: present as fragmented in the new species and absent in *U*. *cirsicola*; (3) and the SIPH L/BL ratio: 0.27–0.32 in the new species and 0.32–0.42 in *U*. *cirsicola* [3,24]. The new species lives on *Asyneuma persicum* and *Michauxia laevigata* in Iran, while *U*. *cirsicola* lives on *Cirsium* spp., *Senecio jacobaea*, and *Tragopogon orientalis* in Ukraine (Crimea), Turkey, and Russia (west Siberia) [3].

The apterous viviparous females of *Uroleucon remaudierei* **sp**. **nov**. are distinguished from those of *U*. *fuchuense* by (1) the number of setae on cauda: cauda with 13–18 setae in the new species, while there are 25–35 setae in *U*. *fuchuense*; (2) the number of secondary rhinaria on ANT III: 7–67 in the new species and 96–135 in *U*. *fuchuense*; and (3) the SIPH L/cauda L ratio: 1.79–2.19 in the new species and 1.10–1.30 in *U*. *fuchuense* [3]. The new species lives on *Asyneuma persicum* and *Michauxia laevigata* in Iran, while *U*. *fuchuense* lives on *Aster* spp. and is also recorded from *Cacalia hastata*, *Pterocypsela raddeana*, and *Saussurea grandifoliai* in Japan, Korea, and Russia (East Siberia) [3,17].

The apterous viviparous females of *Uroleucon remaudierei* **sp**. **nov**. are distinguished from those of *U*. *monticola* by the reticulate area of the SIPH/SIPH L ratio: 0.22–0.35 in the new species and ca. 0.20 in *U*. *monticola* [33]. The new species lives on *Asyneuma persicum* and *Michauxia laevigata* in Iran, while *U*. *monticola* lives on *Aster*, *Coniza*, and *Erigeron* in Japan, Taiwan, China, and Korea [3].

There are fourteen *Uroleucon* species, including a new species living on Campanulaceae (*Adenophora*, *Asyneuma*, *Campanula*, *Jasione*, *Michauxia*, and *Platycodon*), of which four species belong to the subgenus *Uroleucon* and *Uromelan* and remaining ten species belong to the subgenus *Uromelan*. From the Campanulaceae-feeding *Uroleucon*, only two species have a row of short thick peg-like setae on the hind tibia ventrally. They are *Uroleucon adenophorae* and *U*. *campanulae* [11], but both of them belong to the subgenus *Uromelan*, while the new species is a member of the subgenus *Uroleucon*. Differences between all Campanulaceae-feeding *Uroleucon* spp. are given in the identification key.

**Etymology**. We have the pleasure of naming the new species to honor Georges Remaudière—for years an outstanding aphidologist and author of numerous aphid taxa. The name of the species was also the intention of Jaroslav Holman.
**Host plant and biology**. *Uroleucon remaudierei* has been found on two plant species: *Asyneuma persicum* and *Michauxia laevigata* (Campanulaceae). Its sexual morphs and life cycle are unknown.

12. ***Uroleucon (Uromelan) riparium*** (Stroyan, 1955)

*Dactynotus riparius* Stroyan, 1955 [38]: 285

**Apterous viviparous female**. Color in life: dark bronze-brown with black antennae, SIPH, cauda, tarsi, apices of femora, and tibiae [3]. Pigmentation on slide: Head and prothorax sclerotized, brown, antennae dark brown. Femora yellow or pale with dark distal halves (Figure 1k). Tibiae including hind tibiae yellow or light brown with dark knee areas and distal ends (Figure 2k). Peg-like setae very short, robust, with rounded or slightly pointed apices (Figure 3k). Abdomen yellow or pale with well-developed and visible dark scleroites at setal bases, of which the spinal ones on ABD TERG V are larger than the others. SIPH dark brown with well-developed, dark postsiphuncular sclerites. Cauda brown (Figure 4k).
**Host plant**: Species of *Crepis*, *Taraxacum*, and *Tragopogon* (Asteraceae) [3,17].
**Distribution**: In Europe, the species can be found in the northern and central parts (Scotland—*terra typica*, Sweden, Finland, Czech Republic, Slovakia, the European part of the Russian Federation) of Europe [3,17,39] and Kazakhstan [32].

13. ***Uroleucon*** (***Uromelan***) ***siculum*** (Barbagallo and Stroyan, 1982)

*Uroleucon* (*Uromelan*) *ensifoliae siculum* Barbagallo and Stroyan, 1982 [40]: 152

**Apterous viviparous female**. Color in life: dark brown, with mainly black appendages (Blackman and Eastop, 2024). Pigmentation on slide: Head and prothorax sclerotized, brown, antennae brown with slightly lighter ANT III. Femora yellow or pale with dark distal halves (Figure 1l). Tibiae including III TIBIAE light brown with dark knee areas and distal ends (Figure 2l). Peg-like setae short, straight, with clearly pointed apices (Figure 3l). Abdomen yellow or pale with well-developed and visible light brown scleroites at setal bases. SIPH brown with well-developed, brown postsiphuncular sclerites. Cauda brown (Figure 4l).
**Host plant**: Species of *Anthemis*, *Leucanthemum*, *Pulicaria* (Asteraceae), and *Rumex conglomeratus* (Polygonaceae) [3,17].
**Distribution**: This species is mostly known in Sicily in Italy (*terra typica*) and was also recorded in Turkey [41,42].

### 3.2. Notes on the SEM Morphology of Uroleucon remaudierei sp. nov.—Representative of Stridulating Aphids

#### 3.2.1. General Characters

**Figure 8:**
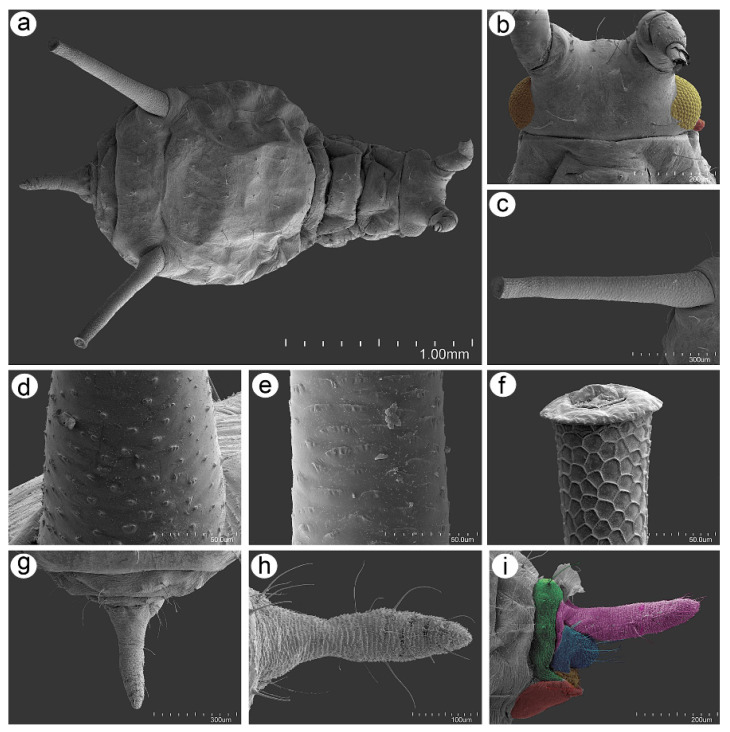
Scanning electron micrographs (SEM) of general morphological characters of the apterous viviparous female of *Uroleucon remaudierei* **sp**. **nov**.: (**a**) dorsal habitus, (**b**) head with compound eyes (yellow) and triommatidia (orange), (**c**) SIPH, (**d**) microsculpture structure of the basal part of the SIPH, (**e**) microsculpture structure of the middle part of the SIPH, (**f**) microsculpture structure of the distal part of the SIPH, (**g**) dorsal side of the end of the abdomen, (**h**) dorsal side of the cauda, (**i**) lateral side of the end of the abdomen showing the perianal structures: ABD TERG VIII (green), cauda (violet), anal plate (blue), rudimentary gonapophyses, (yellow) and genital plate (red).

The body of the apterous viviparous female of *U. remaudierei* **sp**. **nov**. is pear shaped with a wide abdomen. The head is well separated from the pronotum, which is also separated from the rest of the thoracic segments. ABD TERG I–V are fused with separated ABD TERG VII and VIII. SIPH are long, straight, and slightly tapered (Figure 8a). The head is sclerotized with well-developed compound eyes (Figure 8b). The SIPH are imbricated on the whole area (Figure 8c), but the imbrications on the basal part are more protuberant than the sculpture of the middle part of the SIPH (Figure 8d,e). The distal part of the SIPH is characterized by the presence of well-developed polygonal reticulation and flange (Figure 8f). The cauda is finger- or tongue-shaped with short and long setae (Figure 8g–i).

#### 3.2.2. Antennal Sensilla

**Figure 9:**
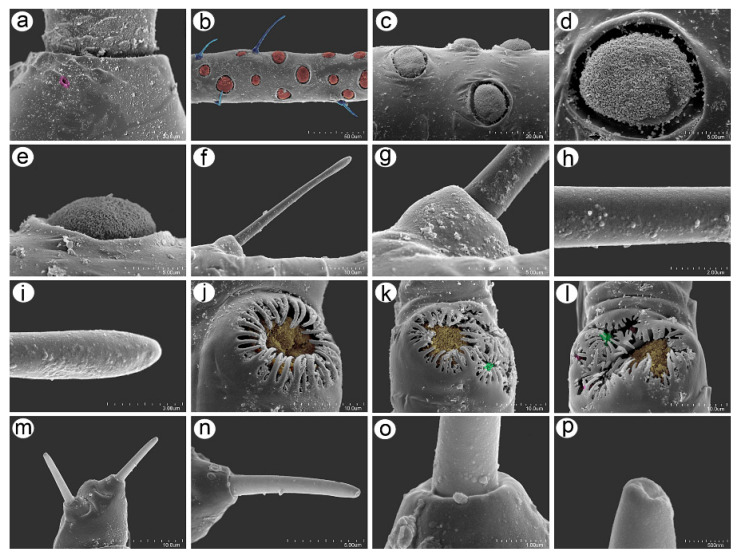
SEM of antennal sensilla of an apterous viviparous female of *Uroleucon remaudierei* **sp**. **nov**.: (**a**) ventral side of pedicel with rhinariolum opening (pink), (**b**) part of ANT III with type I trichoid sensilla (blue) and small multiporous placoid sensilla (red), (**c**) structure of the visibly slightly protuberant small multiporous placoid sensilla on ANT III, (**d**) ultrastructure of the dorsal side of the sensillum showing the sclerotic collar, (**e**) ultrastructure of the lateral side of the sensillum, (**f**) structure of type I trichoid sensillum with the narrow-capitate apical part, (**g**) ultrastructure of the socket of the sensillum, (**h**) ultrastructure of the surface of the sensillum in the middle part, (**i**) ultrastructure of the apical part of the sensillum, (**j**) ultrastructure of the big multiporous placoid sensillum (primary rhinarium, yellow) surrounded with a sclerotic collar with numerous projections on ANT V and (**k**,**l**) sensilla (primary rhinaria) on ANT VI lying and adjoining tightly and surrounded by sclerotic collars with projections: big multiporous placoid sensillum (yellow)—major rhinarium, small multiporous placoid sensilla (green) and poorly-visible sunken coeloconic sensilla —accessory rhinaria, (**m**) type II trichoid sensilla on the apical part of terminal process of ANT VI, (**n**) ultrastructure of the type II trichoid sensillum, (**o**) ultrastructure of the socket of the sensillum, (**p**) ultrastructure of the apical part of the sensillum.

**Figure 10:**
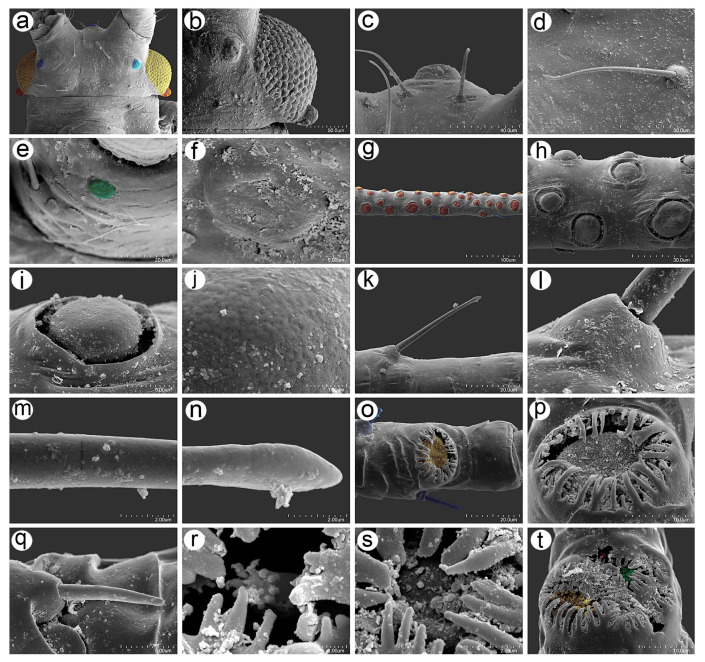
SEM of head and antennal sensilla of an alate viviparous female of *Uroleucon remaudierei* **sp**. **nov**.: (**a**) head capsule with compound eyes (yellow), triommatidia (orange) and ocelli (blue), (**b**) structure of compound eye, triommatidium, and ocellus, (**c**) ultrastructure of the frontal ocellus, (**d**) trichoid sensillum on the head, (**e**) campaniform sensillum (green) on the pedicel, (**f**) ultrastructure of the campaniform sensillum, (**g**) small placoid sensilla (secondary rhinaria) on ANT III (orange), (**h**,**i**) ultrastructure of the small multiporous placoid sensilla, (**j**) ultrastructure of the sensillum membrane with pores, (**k**) type I trichoid sensillum on ANT III, (**l**) socket of the type I trichoid sensillum, (**m**) middle part of the type I trichoid sensillum, (**n**) apex of the type I trichoid sensillum, (**o**) big multiporous placoid sensillum (primary rhinarium) on ANT V (yellow), (**p**) ultrastructure of the big multiporous placoid sensillum, (**q**) type II trichoid sensillum along the terminal process of the ANT VI, (**r**) ultrastructure of the sunken coeloconic sensillum, (**s**) ultrastructure of the small multiporous placoid sensillum on ANT VI, (**t**) group of sensilla on ANT VI with visible big placoid sensillum (yellow), small placoid sensillum (green) and sunken coeloconic sensillum (pink).

The ANT of the apterous viviparous females of *U*. *remaudierei* **sp**. **nov**. bears eight different kinds of sensilla, which can be found on different segments in addition to type I trichoid sensilla, which can be found on all segments. On the ventral side of the pedicel, one small, rounded opening has been found inside, in which the rhinariolum was placed (Figure 9a). On ANT III, in addition to type I trichoid sensilla, small multiporous placoid sensilla of different sizes have been found (Figure 9b). Small multiporous placoid sensilla (secondary rhinaria) are slightly protuberant, and each is surrounded by an evident sclerotic collar (Figure 9c–e). Type I trichoid sensilla on this and other segments are medium in length or long and rigid (Figure 9f); they arise from well-developed sockets at an angle of about 45° (Figure 9g) and have a smooth surface and narrow-capitate apices (Figure 9h,i). On the distal end of ANT V, a big multiporous placoid sensillum (primary rhinarium) has been found, which was lying in the cuticle cavity, surrounded by a sclerotic collar with numerous projections (Figure 9j). On the distal part of the basal part of ANT VI, a group of sensilla (primary rhinaria) has been found. The sensilla are very tightly adjoining to each other and are surrounded by sclerotic collars with numerous projections (Figure 9k). During the examination, a big multiporous placoid sensillum (major rhinarium) was found, together with the rest of the accessory rhinaria on its side—two small placoid sensilla and four sunken coeloconic sensilla (Figure 9l). On the tip of the terminal process of ANT VI, type II trichoid sensilla have been found, which are short and rigid, and have well-developed sockets and blunt apices (Figure 9m–p). Examination of the ANT of the alate viviparous female revealed the same kind and the same characters of the sensilla as in the apterous viviparous female (Figure 10) with an additional sensillum—campaniform sensillum—found on the dorsal side of the alata pedicel (Figure 10e,f).

#### 3.2.3. Mouthparts and Body Sensilla

**Figure 11:**
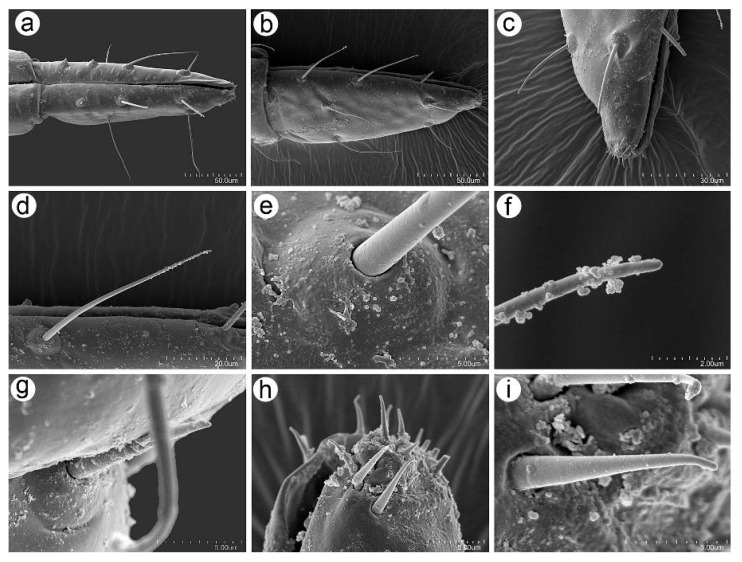
SEM of mouthparts of *Uroleucon remaudierei* **sp**. **nov**.: (**a**) ventral side of the URS with trichoid sensilla, (**b**) lateral side of the URS, (**c**) distal end of the ventrolateral side of the URS with trichoid and basiconic sensilla, (**d**) ultrastructure of trichoid sensillum, (**e**) ultrastructure of the socket of trichoid sensillum, (**f**) ultrastructure of the apex of the trichoid sensillum, (**g**) ultrastructure of the type II basiconic sensillum, (**h**) tip of the URS with type III basiconic sensilla, (**i**) ultrastructure of the type III basiconic sensillum.

The labium (including the URS) bears mostly type I trichoid sensilla, which are long, fine, and pointed (Figure 11a), and the ultimate rostral segments (IV + V) are characterized by the presence of type II basiconic sensilla on the proximal part, often hidden under the third rostral segment and the type III basiconic sensilla (Figure 11b,c). Type I trichoid sensilla on the URS are of the same characters as those on the remaining segments; they are long, fine, and tubular (Figure 11d), arising from flat and rounded, flexible sockets (Figure 11e), and their apices are rounded in higher magnification (Figure 11f). Type II basiconic sensilla also seem to have flexible sockets and are pointed (Figure 11g). Type III basiconic sensilla are distributed only on the very distal end of the URS, are elongate-conical, arising from small cavities in the cuticle, and their apices are slightly rounded (Figure 11h,i).

**Figure 12:**
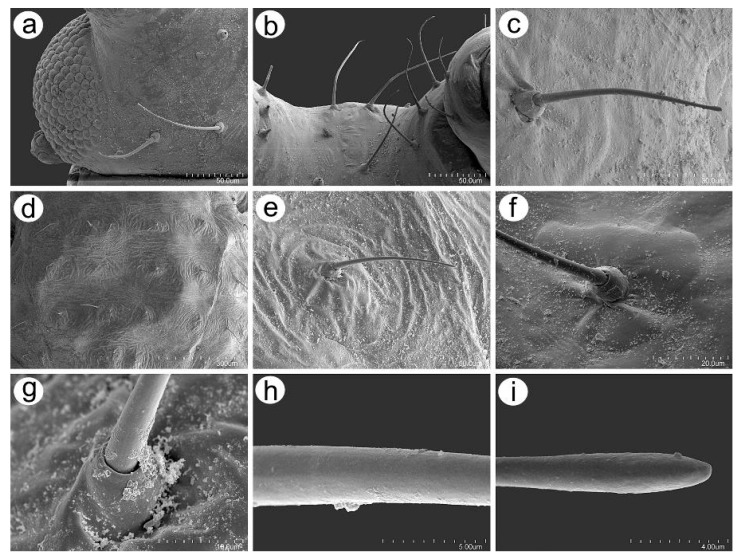
SEM of dorsal body trichoid sensilla of *Uroleucon remaudierei* **sp**. **nov**.: (**a**) sensilla on the head, (**b**) frontal sensilla, (**c**) ultrastructure of the trichoid sensillum, (**d**) abdominal dorsum with sensilla, (**e**) trichoid sensillum on abdominal dorsum, (**f**) ultrastructure of the scleroite at setal base, (**g**) ultrastructure of the socket of the sensillum, (**h**) ultrastructure of the median part of the sensillum, (**i**) ultrastructure of the apex of the sensillum.

The dorsal side of the body, similar to the main part of the labium, is covered by type I trichoid sensilla, which are long and fine and arise from well-developed and flexible sockets (Figure 12a–c). Additionally, type I trichoid sensilla on the dorsal abdomen arise from slightly rounded or irregular in shape scleroites (Figure 12d–f), are tubular and very slightly ribbed near the basal part (Figure 12g), smooth on the middle (Figure 12h), and have narrow-capitate apices (Figure 12i).

#### 3.2.4. Legs Sensilla and the Stridulatory Apparatus

**Figure 13:**
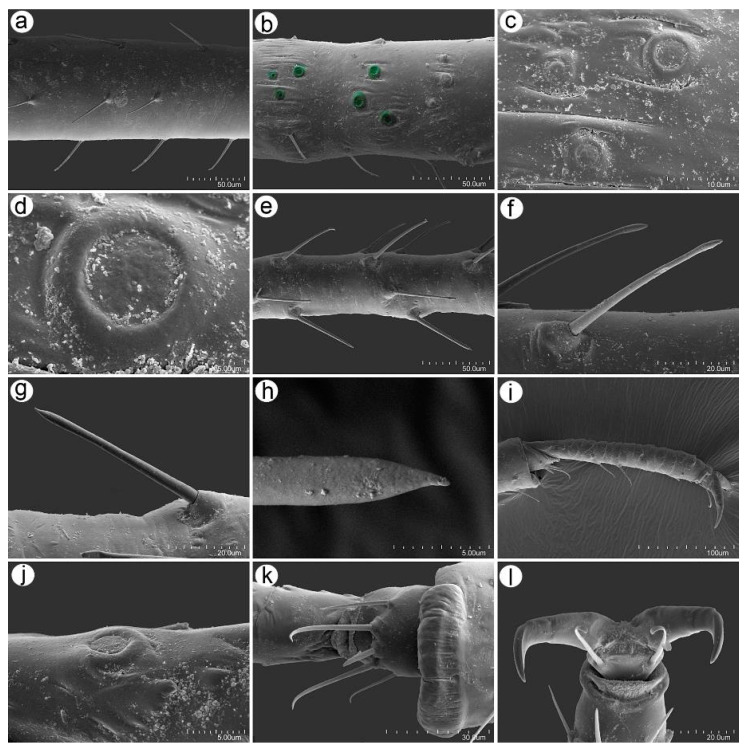
SEM of legs sensilla of *Uroleucon remaudierei* **sp**. **nov**.: (**a**) trichoid sensilla on the femur, (**b**) campaniform sensilla (green) on the trochanter and femur, (**c**,**d**) ultrastructure of the campaniform sensilla, (**e**) trichoid sensilla on the femur, (**f**) structure of the narrow-capitate sensilla, (**g**) structure of the pointed sensilla, (**h**) ultrastructure of the pointed apex of the sensillum, (**i**) hind tarsus with sensilla, (**j**) ultrastructure of campaniform sensilla on HT II, (**k**) sensilla on the ventral side of the HT I, (**l**) HT II parempodia.

The legs of *U*. *remaudierei* **sp**. **nov**. are characterized by numerous trichoid sensilla in the form of medium to long setae, which arise at an angle of about 45° (Figure 13a,e). In addition to the setae on the inner side of trochantera, proximal femora campaniform sensilla have been found (Figure 13b). On trochantera, the campaniform sensilla are of two different sizes (Figure 13c), and their structure is typical with a central pore on the main disc (Figure 13d). Trichoid sensilla on the tibiae are of two different kinds: one type is similar to the sensilla on the ANT and the dorsal side of the body—with narrow capitate apices (Figure 13f). Sensilla of this type are distributed on the proximal part and in the middle of the tibiae. The second type of trichoid sensilla is distributed in the distal area of the tibiae; it is much more rigid and is characterized by straight and pointed apices (Figure 13g,h). The tarsi also bear trichoid sensilla, which are mostly fine and pointed (Figure 13i). The dorsal proximal part of the second segment of tarsi bears one campaniform sensillum (Figure 13j). HT I is characterized by the presence of five sensilla of three different kinds: one central peg-like sensillum, two long sensilla with flattened apices, and two shorter and finer sensilla, also with flattened apices (Figure 13k). Parempodia (empodial setae) are well developed and have clearly flattened and blunt apices (Figure 13l).

**Figure 14:**
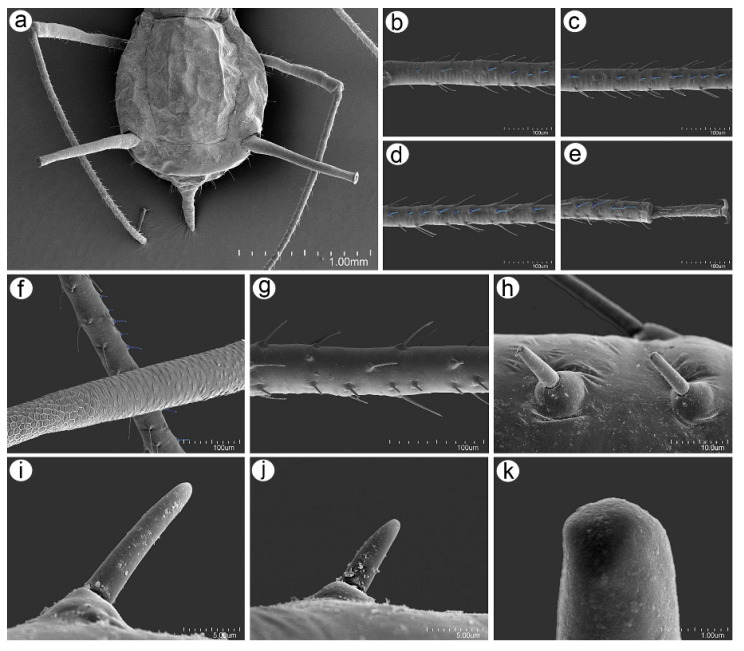
SEM of the stridulatory apparatus of *Uroleucon remaudierei* **sp**. **nov**.: (**a**) the arrangement of the hind legs against the SIPH, (**b**–**e**) row of peg-like setae on the inner side of III TIBIAE, (**f**) the possible ribbing of the III TIBIAE against the SIPH, (**g**–**h**) structure of the peg-like setae, (**i**–**k**) ultrastructure of the apex of the peg-like seta.

The stridulatory apparatus is built from a row of short and very short peg-like setae on the inner side of the III TIBIAE and most probably can be rubbed against the imbricated surface of the SIPH (Figure 14a–f). The distance between the peg-like setae is regular and smaller than the distance between other setae on the tibiae (Figure 14g). The peg-like setae have characters that allow us to classify them as trichoid sensilla, as they have well-visible hemispherical and flexible sockets (Figure 14h). The peg-like setae have two sizes, long and short, are thick, sometimes slightly bulky near the basal side, and have rounded apices (Figure 14i–k).

### 3.3. Identification Key for Campanulaphis and Uroleucon Aphid Species Living on Campanulaceae Based on Apterous Viviparous Females 

This Key is a Modification of Those by Blackman and Eastop [3,12], Including Uroleucon adenophorae, *U. phyteuma*, *U. triphyllae*, and *U. remaudierei* **sp. nov**.

**1.**  Cauda pale or dusky ……………………………………………………………………… **2**

– Cauda dark like SIPH …………………………………………………………………….. **5**

**2.**  URS 0.70–1.07 × HT II ……………………………………………………………………... **3**

– URS 1.10–1.30 × HT II …………………………………………………………………….. **4**

**3.**  Cauda with 13–18 setae. Hind tibia with a ventral row of short thick peg-like setae. On *Asyneuma persicum* and *Michauxia laevigata*. In Iran …………………………………………………………… ***Uroleucon remaudierei* sp**. **nov**.

– Cauda with 9–12 setae. Hind tibia without a ventral row of short thick peg-like setae. On species of *Adenophora*, *Campanula*, and *Platycodon*. In Japan, Korea, and Russia (East Siberia) ………………………………………... ***Uroleucon kikioense*** (Shinji, 1942)*

**4.**  First tarsal segments with five setae. Tibiae wholly dark. ANT III with 50–66 secondary rhinaria. Antesiphuncular sclerites present. On undetermined *Campanula*. In Russia (East Siberia) ………………………………... ***Uroleucon gredinae*** Pashtshenko, 2000

– First tarsal segments with 3 (–4) setae. Tibiae pale on basal 0.7. ANT III with 10–30 secondary rhinaria. Antesiphuncular sclerites absent. On species of *Campanula*. In Tajikistan, Afghanistan, Pakistan, and northern India …………………………………………………… ***Uroleucon kashmiricum*** (Verma, 1966)

**5.**  Large Mtu tubercles present on prothorax and ABD TERG II–IV. On *Asyneuma canescens*. In Slovakia and Ukraine ……………….. ***Uroleucon phyteuma*** (Bozhko, 1950)

– Ordinary Mtu present on prothorax and ABD TERG II–IV …………………………... **6**

**6.**  SIPH 0.85–1.25 × cauda. URS 0.8–1.2 × HT II …………………………………………… **7**

– SIPH 1.5–2.6 × cauda. URS 1.0–2.0 × HT II ……………………………………………… **8**

**7.**  ANT III with 30–60 secondary rhinaria. URS with 8–10 accessory setae. On species of *Adenophora*. In Japan, Mongolia, and Russia (Transbaikalia) …………………………………………………… ***Uroleucon triphyllae*** (Miyazaki, 1966)

– ANT III with 11–35 secondary rhinaria. URS with 4–5 accessory setae. On species of *Campanula* and *Jasione*. In Europe, western Siberia, and southwest and central Asia ……………………………………………… ***Uroleucon campanulae*** (Kaltenbach, 1843)

**8.**  ANT III 2.8–4.4 × URS …………………………………………………………………….. **9**

– ANT III 4.4–6.6 × URS ……………………………………………………………………. **11**

**9.**  Body spindle shaped. URS 1.7–2.0 × HT II. SIPH 5.4–6.4 × HT II. on *Campanula peregrina*. In Lebanon ……………………………………. ***Uroleucon*** sp. (BMNH collection)

– Body oval. URS 1.0–1.45 × HT II. SIPH 2.9–4.2 × HT II ……………………………….. **10**

**10.**  ANT III with 2–18 secondary rhinaria on basal half, and none on ANT IV. On species of *Campanula*. In Italy, Poland, and the former Yugoslavia …………………………………….. ***Uroleucon minosmartelli*** Barbagallo and Patti 1994

– ANT III with 28–45 secondary rhinaria distributed over its entire length, and 0–6 on ANT IV. On species of *Campanula*. In Kazakhstan …………………………………………... ***Campanulaphis radicivora*** Kadyrbekov, 2016

**11.**  Longest setae on outer side of hind tibia 1.5–2.0 × diameter of tibia at midlength. Longest setae on ANT III 1.2–1.5 × BD III. On species of *Campanula* and *Platycodon grandiflorus*. In Japan, Kazakhstan, and Russia (East Siberia) ……………………………………………. ***Uroleucon neocampanulae*** (Takahashi, 1962)

– Longest setae on outer side of hind tibia 0.9–1.2 × diameter at midlength. Longest setae on ANT III 0.8–1.3 × BD III ……………………………………………………….. **12**

**12.**  ANT PT 5.0–6.2 × ANT VI BASE. ANT III 5.7–7.1 × ANT VI BASE ………………… **13**

– ANT PT 6.2–8.3 × ANT VI BASE. ANT III 7.0–8.0 × ANT VI BASE ………………… **14**

**13.**  Cauda with 11–14 setae. Longest setae on ABD TERG III–V are at least 2 × BD III. On unidentified *Campanula*. In France ……………………………………………………………………………………… ***Uroleucon ariegense*** Nieto Nafría and Pérez Hidalgo, 2013

– Cauda with 14–19 setae. Longest setae on ABD TERG III–V 1 or slightly longer × BD III. On species of *Adenophora.* and *Astrocodon kruhseanus*. In Japan, Mongolia, and Russia (Transbaikalia) ……………………… ***Uroleucon adenophorae*** (Matsumura, 1918)

**14.**  ANT III with 19–58 secondary rhinaria at a density of 20–58 per mm, extending over 0.42–0.94 of the segment. (Al. with 43–78 secondary rhinaria on ANT III and none on ANT IV.) On species of *Campanula*. In Europe and southwest and central Asia ………………………………………………….. ***Uroleucon rapunculoidis*** (Börner, 1939)

– ANT III with 52–122 secondary rhinaria at a density of 45–84 per mm, extending over 0.77–0.97 of the segment. (Al. with 97–137 secondary rhinaria on ANT III, and usually without but sometimes with 1–7 on ANT IV.) On species of *Campanula*. Widely distributed in Eurasia …………………… ***Uroleucon nigrocampanulae*** (Theobald, 1928)

* Modification for *Uroleucon kikioense* was performed using the redescription given in Pashtshenko (2001).

### 3.4. Key to Apterous Viviparous Females of the Known Sound-Producing Species of the Genus Uroleucon

**1.**  Cauda pale or yellow …………………………………………………………………….. **2**

– Cauda dark ………………………………………………………………………………... **7**

**2.**  Abdomen with large marginal tubercles ……………….. ***U. phyteuma*** (Bozhko, 1950)

– Abdomen without large marginal tubercles …………………………………………… **3**

**3.**  Abdomen without dark, well-visible scleroites at setal bases ………………………….…………………………………………………………… ***U. monticola*** (Takahashi, 1935)

– Abdomen with dark, well-visible scleroites at setal bases …………………………… **4**

**4.**  Abdomen with light brown scleroites at setal bases and poorly developed and poorly visible postsiphuncular sclerites ………………………………………………... **5**

– Abdomen with dark brown scleroites at setal bases and dark, well-developed postsiphuncular sclerites ………………………………………………………………… **6**

**5.**  SIPH no longer than 1.2 × cauda, secondary rhinaria on ANT III distributed on about ¾ of the length of the segment ……………………... ***U. fuchuense*** (Shinji, 1942)

– SIPH longer than 1.2 × cauda, secondary rhinaria on ANT III distributed only to ½ of the length of the segment ……………….. ***U. capsicum*** Rezwani and Lampel, 1990

**6.**  Abdomen with few scleroites at setal bases, cauda with 13–18 setae ………………….…………………………………………………………………….. ***U. remaudierei* sp. nov.**

– Abdomen with many scleroites at setal bases, cauda with 30–45 setae ………………..………………………………………………………………... ***U. cirsicola*** (Holman, 1962)

**7.**  SIPH not more than 1.5 × cauda ………………………………………………………… **8**

– Siph more than 1.5 × cauda ……………………………………………………………… **9**

**8.**  Abdomen with antesiphuncular sclerites absent ………………………………………………………………………………………………. ***U. adenophorae*** (Matsumura, 1918)

– Abdomen with antesiphuncular sclerites present ……………………………………………………………………………………………….. ***U. campanulae*** (Kaltenbach, 1843)

**9.**  Hind tibiae uniformly dark ………………………………… ***U. jaceae*** (Linnaeus, 1758)

– Hind tibiae at least with some lighter part …………………………………………… **10**

**10.**  Abdominal scleroites in spinal and pleural area all of the same size ……………………………………………………………………… ***U. carthami*** (Hille Ris Lambers, 1948)

– Abdominal scleroites in the spinal area are larger than those in the pleural area ….. **11**

**11.**  Abdominal spinal setae as long as or shorter than the scleroites width, scleroites on ABD VII are fused into larger sclerites ………………………. ***U. minor*** (Börner, 1940)

– Abdominal spinal setae longer than the scleroites width, scleroites on ABD VII not fused into larger sclerites ……………………………………………………………….. **12**

**12.**  Abdomen with many pleural scleroites, mesothoracic furca sesille …………………………………………………………………………………. ***U. riparium*** (Stroyan, 1955)

– Abdomen with few pleural scleroites, mesothoracic furca with elongate stem ………………………………………………………… ***U. siculum*** Barbagallo and Stroyan, 1982

## 4. Comments

The stridulation phenomenon, in fact, has been reported and confirmed only in *Aphis aurantii* by records of the produced sound [43]. It is most probable that other species of the *Aphis* subgenus *Toxoptera* (*A. citricida* and *A. odinae*), which, in addition to short peg-like setae on the legs, have a reticulate pattern on their abdomen [9], also can produce sound. Our study is the very first of planned research on the possible sound-producing structures in *Aphis* (*Toxoptera*) and other Macrosiphini (*Macrosiphoniella* and *Uroleucon*), including morphological and ultrastructural analyses. The first examination of this apparatus in *Uroleucon remaudierei* clearly shows that the sound in *Uroleucon* species (and most probably in *Macrosiphoniella*) is produced not by the rubbing against the abdomen but against the siphunculi, whose surface is evidently imbricated, and the apical part has a well-developed reticulation. As the possible sound is produced in another way, for sure, the rhythm pattern and the spectrum should be completely different, as known in *A. aurantii* [43]. Continuing, as the sound in both groups of the morphological apparatus is different, its importance should be treated as distinct. Of course, there are no studies that could definitely define the sense and meaning of the sound produced by *A. aurantii*, and we can only suspect that the sound is used for defense, masking, or to help find the colonies by the ants. The more the sense and significance of the sound-producing apparatus discovered by Holman [11] and described using SEM for the first time in this study may be different and detailed analyses, including morphological and molecular approach, will for sure help to elucidate the eventual meaning of the characters of the peg-like setae on the tibiae in particular species.

Our review of a group of species belonging to the genus *Uroleucon* that have a peculiar characteristic, peg-like setae on their hind tibiae, may give rise to the apparent paradox that they belong to two subgenera (*Uroleucon* and *Uromelan*). In addition, two botanical families were involved as host plants for the group. This may lead to a basic question—are the sound-producing structures of taxonomical or phylogenetic significance? If yes, perhaps it is one more reason to reflect on the artificiality of the subgeneric division of *Uroleucon*, which has been put forward by various authors for the last thirty years at least. On the other hand, a deeper examination of the stridulatory apparatus of *Uroleucon* (and other Macrosiphini) may show that in the case of this group, depending on its primary importance, the structures evolved independently and are solely responsible only for responding to different needs resulting from different ecological and biological impacts.

## 5. Conclusions

A detailed and comparative review of the stridulating species of the aphid genus *Uroleucon* showed that aphid morphology still needs to be investigated for a better understanding of the overall biology of this group. Also, a deeper analysis of the genus *Uroleucon* is crucial to know the level of diversity of aphids in this genus, their taxonomy, and phylogenetic relationships, and this is the first and second authors’ long-term and ongoing research.

## Figures and Tables

**Table 1:** Measurements (in mm) of apterous and alate viviparous females of *Uroleucon remaudierei* **sp**. **nov**.

Character	Apterous Viviparous Female	Alate Viviparous Female
BL	2.22–3.32	2.80–3.12
HW	0.45–0.56	0.48–0.53
ANT	2.34–3.06	2.75–3.23
ANT III	0.61–0.87	0.80–0.95
ANT IV	0.42–0.61	0.50–0.66
ANT V	0.35–0.45	0.38–0.51
ANT VI	0.74–0.99	0.81–0.85
BASE	0.15–0.16	0.16–0.17
PT	0.58–0.83	0.65–0.68
URS	0.15–0.18	0.17–0.18
FEMORA III	0.80–1.15	1.00–1.20
TIBIAE III	1.50–2.2.	2.05–2.35
HT II	0.15–0.18	0.18–0.20
SIPH	0.65–0.92	0.85–1.07
CAUDA	0.36–0.50	0.35–0.43

## Data Availability

All data generated or analyzed during this study are included in this published article.
